# Minimally Invasive Treatment of Lateral Incisors with Guided One-Piece or Two-Piece Titanium-Made Narrow Diameter Implants: A Retrospective Comparative Study with Up to Two Years Follow-Up

**DOI:** 10.3390/jcm12113711

**Published:** 2023-05-27

**Authors:** Łukasz Zadrożny, Bartłomiej Górski, Edoardo Baldoni, Aurea Immacolata Lumbau, Silvio Mario Meloni, Milena Pisano, Marco Tallarico

**Affiliations:** 1 Department of Dental Propaedeutics and Prophylaxis, Medical University of Warsaw, Nowogrodzka 59 St., 02-006 Warsaw, Poland; 2 Department of Periodontology and Oral Mucosa Diseases, Medical University of Warsaw, Binieckiego 6 St., 02-097 Warsaw, Poland; bgorski@wum.edu.pl; 3 Department of Medicine, Surgery, and Pharmacy, University of Sassari, 07021 Sassari, Italy; baldoni@uniss.it (E.B.); alumbau@uniss.it (A.I.L.); melonisilviomario@yahoo.it (S.M.M.); milenapisano@yahoo.it (M.P.); me@studiomarcotallarico.it (M.T.)

**Keywords:** narrow diameter implants, one-piece implant, two-piece implants, NDI, esthetics, PES, MBL

## Abstract

Restoring teeth with dental implants has become the gold standard in recent years, especially in the esthetic zone. However, limited amount of available bone as well as limited interdental space in the anterior zone may create problems for implant treatment. Narrow diameter implants (NDI) may be a treatment option to resolve the above-mentioned limitations and providing minimally invasive implant therapy without additional regenerative procedures. In this retrospective study, a comparison of clinical and radiographic outcomes between one-piece and two-piece titanium-made NDIs was done with the follow-up of two years after loading. Twenty-three NDI cases were analyzed, 11 in the one-piece implant group (group one) and 12 in the two-piece implant group (group two). The outcomes were implant and prosthetic failures, any complications occurred, peri-implant bone level changes, and as well as the Pink Esthetic score. No implant or prosthetic failures, as well as, no complications were reported at the two-year follow-up examination. At the same time the marginal bone loss was 0.23 ± 0.11 in the group one and 0.18 ± 0.12 in the group two. Difference was not statistically significant (*p* = 0.3339). The Pink Esthetic Score, recorded two years after definitive loading, was 12.6 ± 0.97 in the group one and 12.2 ± 0.92 in the group two, with no statistically significant difference between groups (*p* = 0.3554). With the limitations of the present study, including the small sample size and short follow-up, it is possible to conclude that either one and two-piece NDI can be successfully used to restore lateral incisors with comparable results within the two years of follow-up.

## 1. Introduction

The oral cavity is a limited area of the human body; however, oral health widely influences the general health of people [1,2,3]. In addition, damaged dentition and/or lack of teeth lead to functional, esthetic and social problems. Edentulism especially in the anterior area or so called esthetic zone may lead to a decrease in self-confidence and even mental problems [4,5]. In 2010, almost four billion people were affected by oral conditions, with the burden increasing by 20.8% over the two previous decades [6]. Different bone defects, caused by trauma, infections, parafunctions or cancer affecting individuals of all ages, result in an enormous financial burden across the world. Globally, the direct annual costs of dental diseases reached $356.8 and the estimated productivity loss due to oral diseases was $187.6 billion in 2015 [7]. Soft and hard tissue loss due to periodontitis and severe edentulousness are the major contributors to this burden of disease [8], and regeneration of the lost tissues is considered the ultimate treatment outcome. Congenital tooth agenesis is also a dental anomaly affecting the esthetic zone, with reported incidences of 2.7% to 12.2%, excluding the third molars [9,10]. In the permanent dentition, maxillary lateral incisors are the most commonly affected, with a prevalence rate between 1% and 4% [8]. Treatment options for missing lateral incisors include space opening, followed by the placement of a conventional fixed bridge or a single-unit implant-supported crown, and orthodontic space closure with anatomical recontouring of the canines [10]. However, treatment with dental implants is today considered the gold standard approach in several situations, achieving long-term survival and success rates [11,12]. Nevertheless, aesthetic rehabilitation, as well as treatment of atrophic jaws, are classified as high-risk procedures [5,13]. To overcame the risk of implant failure or aesthetic complications, guided bone regeneration may be recommended to achieve successful long-term results [14,15,16]. For the latter, clinicians, as well as patients, still consider as alternative treatment options, several procedures including Maryland bridge, conventional teeth supported bridge, and short or narrow implants [17,18,19,20,21].

In the last years, through the increased demand of minimally invasive approaches, narrow diameter implants (NDIs) became more popular. According to a recent systematic review with a meta-analysis of Eik Schiegnitz and Bilal Al-Nawas [21], narrow diameter implants were classified into Category 1 (implant diameter < 3.0 mm, “mini-implants”), Category 2 (implant diameter 3–3.25 mm) and Category 3 (implant diameters 3.3–3.5 mm). Usually, NDIs are one-piece implants; however, two-piece NDIs were introduced recently, with an aim to address some prosthetic problems and to provide more prosthetic options. Both implants demonstrated effectiveness in the rehabilitation of patients requiring dental implants to retain and implant-supported restoration [21]. Nevertheless, there are no data that allows to compare one- versus two-piece narrow diameter implants for the treatment of incisors

The aim of the present study is to retrospectively compare clinical and radiographic data of patients treated with one-piece versus two pieces titanium-made narrow diameter implants for the treatment of upper lateral and lower incisors with a follow-up period of at least 2 years after loading.

## 2. Materials and Methods

This observational study was designed as a retrospective comparative evaluation, aimed to understand whether is preferable to use one or two-piece narrow diameter implants for the rehabilitation of the upper lateral and lower incisors.

### 2.1. Patient Selection

A cohort of patients, aged at least 18 years, who received at least one NDI to support a single crown in the maxillary lateral or mandible incisors, with at least two years of follow-up and available peri-apical radiographs taken at implant placement, loading, one- and two-year after, were considered for this study. The study was performed according to the principles of the Helsinki Declaration published in 2013. Patients were informed about all materials and clinical procedures, benefits, potential risks and complications. Written informed consent was obtained for all performed procedures. All medical data were anonymized so that patient cannot be identified. Ethics clearance was granted by the Ethical Committee by the Medical University of Warsaw, Poland. Approval code: AKBE/41/2023.

Exclusion criteria were as follows: no available radiographs and signed informed consent, patients who underwent guided bone regeneration, immediate implants (the socket sites had to heal for four months), heavy smokers (≥11 cigarettes per day), local acute or chronic infections at the time of implant placement, poor oral hygiene (bleeding on probing and/or plaque index > 25%), absence of teeth in the opposite jaw, and severe bruxism or jaw clenching.

A retrospective chart review of data collected in two centers (Poland and Italy), including documents, radiographs, and clinical pictures, was performed by an independent examiner (MP). Patients were grouped based on their implant abutment interface and diameter. In group 1, one-piece NDI of 2 or 2.5 mm of diameter (Category 1 or mini implants) and 10 to 13 mm of length were positioned. In group 2, two-piece NDI of 3 or 3.5 mm of diameter (Category 2 and 3) and 10 to 13 mm of length were placed. The rationale to choose one- or two-piece implants as well the diameter size was to allow a prosthetically driven position of the implants using a minimally invasive approach.

### 2.2. Surgical Protocol

All the implants came from the same manufacturer (Osstem Global Co., Ltd., Seoul, Republic of Korea) and presented the same SA (sandblasted and acid-etched) surface. The main difference is that in the one-piece implants (Osstem MS Implants, Osstem Global Co., Ltd.), the fixture and the abutment portions consist of a single unit. On the contrary, two-piece implants (Osstem TSIII, Osstem Global Co., Ltd.) consist in two separate portions. Both TSIII implants of 3.0 and 3.5 mm of diameter, presented the same internal, conical, implant abutment interface, with internal hex and the 11° taper connection. MS Implants and TSIII of 3.0 mm diameter are made of titanium grade V, while TSIII implants of 3.5 mm diameter are made of grade IV.

Narrow diameter implants were placed according to the manufacturer instructions. All surgical and prosthetic procedures were performed by two experienced oral surgeons (LZ and MT) using a computer-assisted, template-based approach. All the implants were placed flapless or with a minimally invasive flap (without vertical incisions) depending on the amount of keratinized tissue, using a computer-assisted, template-based approach. Surgical templates were designed without metallic sleeves for surgical kits dedicated to TSIII or MS implants (OneGuide Kit or OneMS Kit, Osstem Global Co., Ltd.) [22]. Implants were placed in a prosthetically driven position, according to a pre-established wax-up. In group one, the implants were placed slightly palatal in order to have the screw hole at the cingulum of the restored teeth. In group 2, the implants position was planned in order to have the abutment inside the volume of the teeth to be rehabilitated. Two-piece implants were placed at a bone level of slightly deeper in order to have 4 mm for the emergence profile of the crowns [23,24,25,26,27,28,29,30]. All the implants were planned maintaining at least 1.5–2 mm of the buccal bone, without any bone and/or soft tissue augmentation procedures. The maximum insertion torque was 30 N cm (according to the manufacturer instructions). Immediate loading was performed if the insertion torque was at least 25 Ncm, measured with the surgical micromotor (Surgical Pro, Nakanishi Inc., Kanuma, Japan). In order to avoid any static and dynamic contacts, all of the implants received a non-functional temporary restoration. In case of delayed loading, the temporary restoration was placed after three months of healing.

### 2.3. Prosthetic Protocol

**Figure 1:**
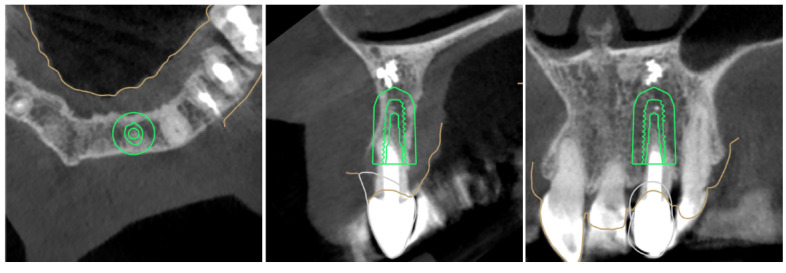
Digital planning process, two-piece implant, 3.5 × 10 mm TSIII SA to replace tooth 22.

**Figure 2:**
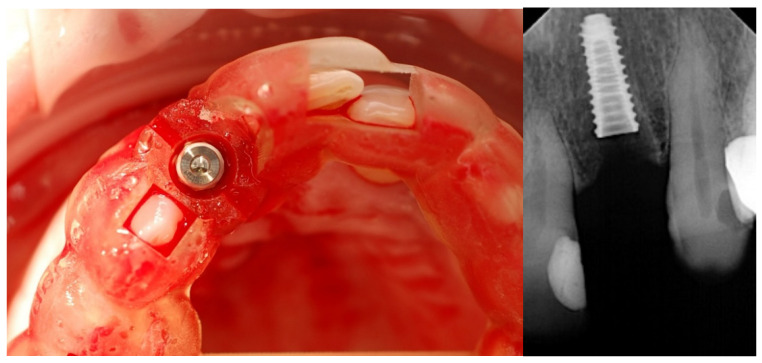
Guided implant placement.

**Figure 3:**
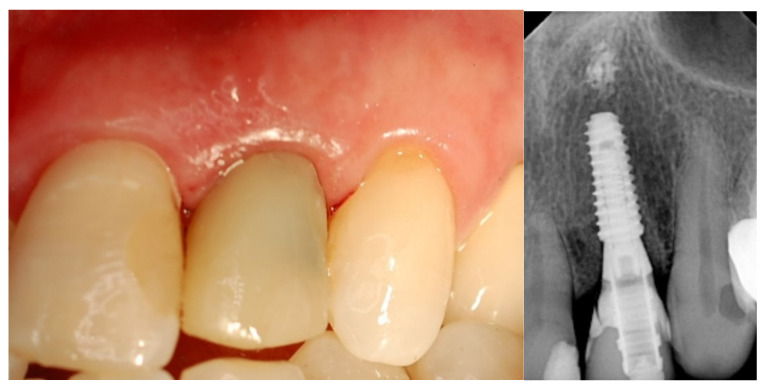
Immediate loading screw-retained crown.

**Figure 4:**
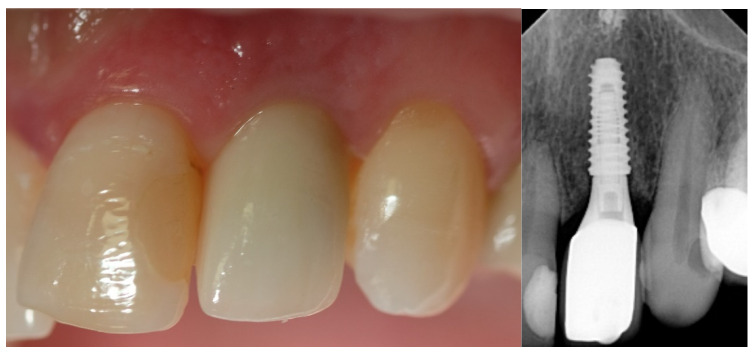
Final crown delivery.

**Figure 5:**
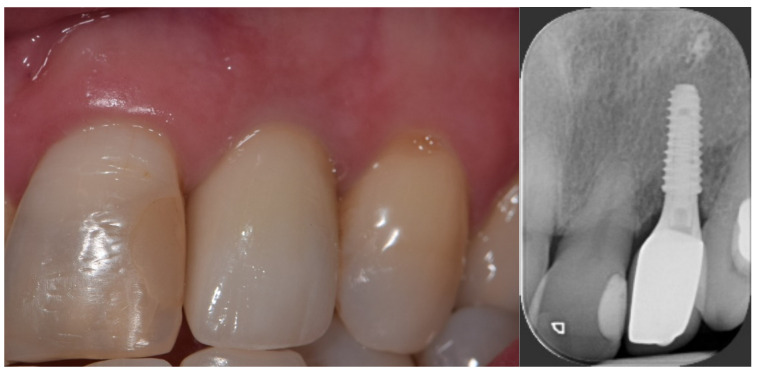
At two years of follow-up.

**Figure 6:**
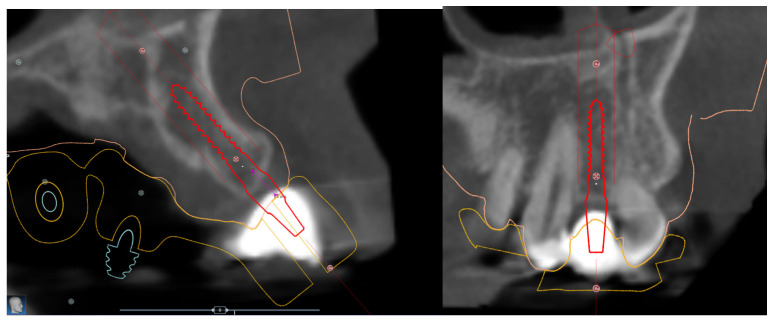
Digital planning process, one-piece implant, 2.5 × 11.5 mm MS SA to replace tooth 22.

**Figure 7:**
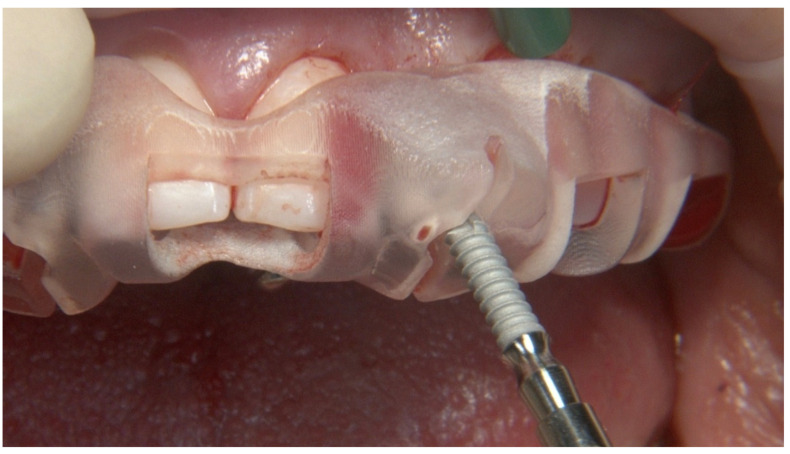
Guided implant placement of one-piece implant.

**Figure 8:**
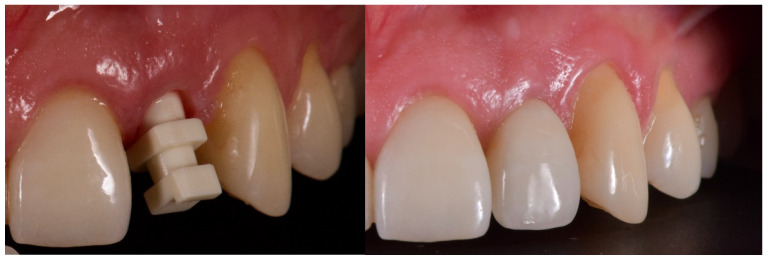
Impression with transfer coping and cemented temporary crown-immediate loading.

**Figure 9:**
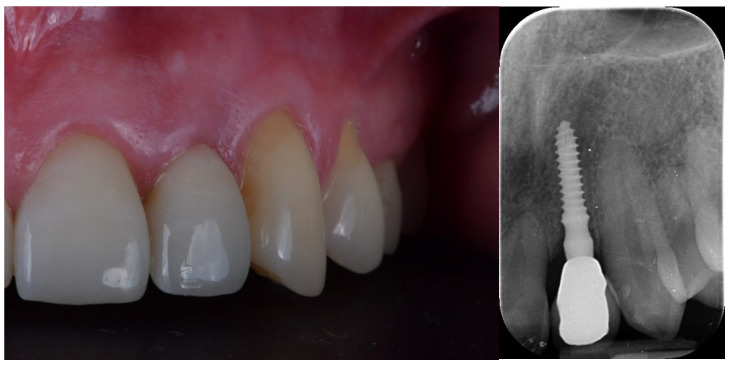
Final crown delivery.

**Figure 10:**
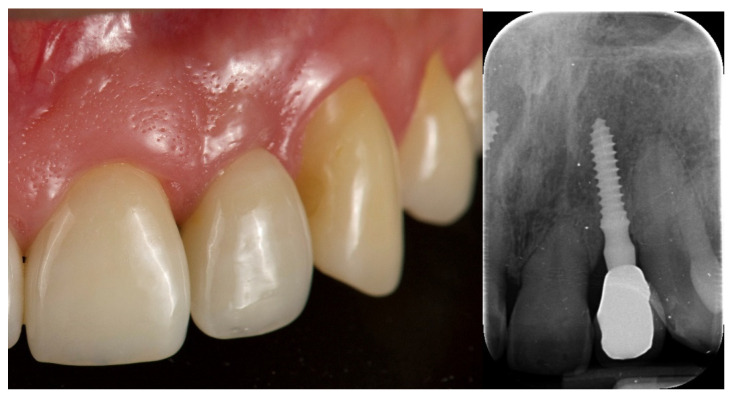
At two years of follow-up.

Three to four months after temporary restoration delivery, a definitive impression was taken, and porcelain fused to zirconia restorations were delivered within four weeks. In group 1, the definitive restorations were cement-retained (Ketac Cem, 3M ESPE AG, Seefeld, Germany), while, in group 2, screw-retained restorations bonded on the base abutments (Osstem Global Co., Ltd.) were delivered. Figure 1, Figure 2, Figure 3, Figure 4 and Figure 5 present the whole protocol of exemplary cases with two-piece implants, and Figure 6, Figure 7, Figure 8, Figure 9 and Figure 10 present an exemplary case with one-piece implants.

### 2.4. Outcome Measures

Outcome measures were as follows: implant and prosthetic failures, any complications, and marginal bone level changes.

- Implant failure was defined as mobility, infection, fracture, and/or any other mechanical or biological problem that determined implant removal.
- A prosthesis was considered a failure always if it had to be replaced;
- Any biological complications (e.g., pain, swelling, mobility, suppuration) and/or technical issues (e.g., material fractures, screw loosening) were reported during follow-up;
- Esthetic evaluation of clinical pictures, including at least two adjacent teeth, taken at two years after loading was done following the pink esthetic score (PES) proposed by Fürhauser et al., in 2005 [31]. In brief, the PES score evaluates seven variables: mesial papilla, distal papilla, soft tissue level, soft tissue contour, alveolar process deficiencies, soft tissue color and texture. A 0-1-2 scoring system was used, with a maximum achievable score of 14 per site.

Peri-implant bone levels were measured at the mesial and distal margins of the implants. In the one-piece implants, the marginal bone level was measured as the distance between the most coronal point where the bone comes in contact with the implant (bone-to-implant contact) and the underside of the gingival portion. In the two-piece implants, the marginal bone level was measured as the distance between the most coronal point where the bone comes in contact with the implant (bone-to-implant contact) and the most coronal portion of the implant platform. Marginal bone loss was calculated as the differences between marginal bone levels at different timepoints. All the measurements were made on digital periapical radiographs, taken using the parallel technique with an extension cone paralleling instrument (Rinn XCP, Dentsply, Elgin, IL, USA). All radiographs and measurements were analyzed with DFW 2.8 software for MS Windows (Soredex, Tuuka, Finland), calibrated for each image separately using the known implant length or diameter. A dental specialist (AIL), previously not involved in this study, performed all radiographic measurements.

### 2.5. Statistical Analysis

Differences between groups were compared with the Fisher exact probability test for dichotomous variables (for implant and prostheses failures and complications). The Mann–Whitney U tests was used for continuous variables (for peri-implant bone levels). A level of significance of 0.05 was applied to all conducted analyses. Implants were used as the statistical unit, and statistical analyses were performed using SPSS for Mac OS X ver. 22.0 (IBM, Chicago, IL, USA).

## 3. Results

A total of 26 medical records of patients who received narrow diameter implants were found. Of these, five patients were excluded. Two patients received two two-piece 3.0 mm diameter implants in combination with guided bone regeneration. Other two received 3.5 mm implants combined with the socket shield procedure. The fifth patient receive a one-piece 2.5 mm implant to support a single crown in the canine position. Data from 21 other patients (11 women, 10 men; range 19–81 years old; average age 55.6 years) were selected according to the inclusion and exclusion criteria. Two patients, one in each group, received two implants (bilateral agenesis of the upper lateral incisors). Finally, the data from 23 (11 in group one and 12 in group two) implants were evaluated. All the implants were placed flapless or with a mini flap without vertical incisions, subcrestally, according to the manufacturer recommendations. Patient and implant characteristics are reported in Table 1. There are no statistically significant differences between groups, except for a longer follow-up in group 2. Nevertheless, data were reported at the same follow-up period of 2 years after definitive loading.

The pink esthetic score recorded two years after definitive loading was 12.6 ± 0.97 in group 1 and 12.2 ± 0.92 in group 2, with no statistically significant difference between groups (*p* = 0.3554).

At the two years follow-up examination, the marginal bone loss was 0.23 ± 0.11 in group 1 and 0.18 ± 0.12 in group 2. The difference was not statistically significant (*p* = 0.3339).

No implant or prosthodontic failure as well as other complication were reported at the two-year follow-up.

## 4. Discussion

The one-piece implants were originally designed to address the structural weakness issues that were part of the two-piece implants. Moreover, in order to improve the mechanical proprieties, some companies manufacture narrow diameter implants using titanium grade V, as in this research. In the last decade, patient preference for minimally invasive treatment options that exclude bone augmentation is improved [31,32]. However, treatment of missing incisors may be based on different possibilities including conventional or adhesive bridges with good results even in almost ten years follow-up [20]. Nevertheless, implant-based reconstructions especially in the esthetic area becoming a gold standard with acceptable results in terms of both esthetic and function with follow-up reaching almost twenty years [12].

According to the results of a recent systematic reviews, narrow diameter implants of less than 3.0 mm of diameter (Category 1) performed statistically significantly worse compared with wider NDIs that were suggested for the rehabilitation of limited interdental spaces in anterior single-tooth restorations [21].

In the present research, no differences were found between tested implants. A possible explanation could be that all the used implants were placed in a prosthetically driven positions, in the native bone, and with at least 1.5 mm of the buccal bone. Moreover, 2 to 3 mm of diameter implants were made of titanium grade V, with similar resistance to implants with wider (3.5 mm) diameter. Finally, all the implants are the same modified surface (sand-blasted and acid-etched)

Narrow diameter implants of 3.5 mm of diameter were described in all regions, including posterior single-tooth restorations [33,34]. However, certain conditions must to be respected, including stronger implant material as titanium grade V [35].

In the present study, all the implants were placed according to a minimally invasive approach, using a surgical template and avoiding bone and soft tissue augmentation. The NDIs can be used when guided bone regeneration is not indicated or in case patients refused invasive surgical procedures.

When planning the treatment with one-piece NDIs, it is important to take into account some prosthetic factors. One-piece implants only allow for cemented retained restorations, and when used in narrow, mesio-distal, spaces, esthetics may be a challenge. Basing on the available literature, it is well known that today computer-assisted, template-based surgery is beneficial to place the implants in the correct position providing satisfied aesthetic results [35,36,37,38]. The accuracy of guided surgery is well known [22,39,40], especially, in a single tooth missing in the anterior area.

Implant depth is another than position and angulation factor crucial for bone stability around implants after prosthetic reconstruction. The depth of implant placement should be carefully planned accordingly to the available bone and soft tissue thickness, the type of the implant, implant-abutment interface and the type and design of further prosthetic reconstruction. Particularly, not the depth of the implant placement but the distance between the cervical margin of planned restoration and the implant platform is the key factor providing a biological space, named the transitional zone, for peri-implant soft tissues and biologic width establishment [24,25,27,28].

Considering all guided approach advantages, we cannot forget about planning phase when all factors such as the implant type, implant-abutment interface, position, angulation and distance between future prosthesis and implant platform can be evaluated and backward planned to fill above mentioned dimensional biological requirements. The peri-implant space, or in other words, the implant supra crestal complex may be important to obtain successful long-term results for implant treatment [41]. Proper implant positioning together with an appropriate implant supra crestal complex provide biological conditions to prevent marginal bone loss, thus resulting in good esthetic results e.g., measured with PES.

Within two years of observation, we have not found differences between the tested groups in all the tested outcomes; however, all cases were carefully planned by experienced clinicians. Regarding the peri-implant soft tissue space, we planned to use about four-millimeter high abutments to restore two-piece implants and four-millimeter distance between the margin of the crown cemented on the one-piece implants and the implant thread.

Kraus et al., in a RCT with five years of follow-up, concluded that cemented restorations on implants were associated with a higher biological and overall complication rate. However, these authors found a relatively low survival rate for both tested prostheses as well as cemented screws retained [42]. Moreover, they did not compare one- and two-piece implants as we did in this study.

There is still no consensus in the literature if cemented or screw retained prostheses have higher overall success rates. Different meta-analyses point out that the problem is that no standardized criteria are available, making them difficult to compare [43,44]. Furthermore, standardized studies with long follow-up are needed to resolve this issue and set clinically relevant conclusions for practitioners.

In the present research, patients with bruxism or severe clenching were excluded. Although bruxism is quite common, it should be considered as a risk factor for any dental treatment and may lead to implant failure, and mechanical complications for implant-supported prosthesis as well to teeth supported prostheses chipping, or adhesive restorations debonding [45].

The limitations of our study are the retrospective nature and a relatively small group. Another limitation is that the patients were followed-up for only two years, giving the presented results. However, the compared groups are even. These authors will continue the observation of the collected group to obtain longer follow-up data. According to Tecco et al., patients will be monitored for periodontal indicators, such us plaque index, bleeding on probing, and probing pocket depth, in order to further understand the role of different behaviors of one- versus two-piece implants, and their role on secondary implant failure due to peri-implantitis [46]. Another limitation is that this study design with completely the same implant surface allowed for only two different collar designs to be compared. However, from the literature we know that different implant collar designs as well as different internal connections may influence the outcomes of implant treatment in terms of bacterial microleakage and peri-implant bone stability [47,48,49]. Then, on the basis of these results, we see the need for further studies comparing one-piece and two-piece implants with different implant-abutment connections and different thread designs in terms of esthetic and biological performance. Then, different implants restored with screws retained and cemented prostheses performed with the respect of biologic dimensions (abutments height) required for healthy peri implant tissues. Moreover, as we see advantages of proper implant positioning with the guided approach according to prosthetic plan containing the implant position and angulation and depth, resulting in the need of using appropriate heights of the prosthetic abutments.

## 5. Conclusions

With the limitations of our study, we can conclude that either one and two-piece NDIs can be successfully used to restore upper and lower lateral incisors with comparable results within the two years of follow-up. However, it was not based directly on the results of this study, but all presented cases were performed considering the digital approach; thus, we conclude that the proper planning and positioning of dental implants may be the crucial factor for functional as well as the esthetic results of implant treatment. A longer observational study may be beneficial to support these preliminary findings.

## Figures and Tables

**Table 1:** Patient and implant characteristics between groups.

Data	One-Piece	Two-Piece	*p*-Value
Number of patients/implants	10/11	11/12	1.0
Mean age ± SD ^1^ (range)	36.5 ± 19.4 (19–81)	29.7 ± 12.8 (19–85)	0.3437
Male/Female	4/6	7/4	0.3949
Total follow-up in months	28.5 ± 3.4 (24–34)	38.7 ± 10.5 (24–60)	0.0095
Implant diameter	2 or 2.5 mm	3 or 3.5 mm	NA ^2^
Implant length	10 to 13	10 to 13	NA ^2^
Crowns	Cemented-retained	Screw-retained	NA ^2^
Maxillary/mandible implants	10/1	11/1	1.0
Immediately/delayed loaded	11/0	9/2	0.4762
Implant failure	0	0	1.0
Prosthetic failure	0	0	1.0
Complications	0	0	1.0
PES ^3^ at the 2-year follow-up	12.6 ± 0.97	12.2 ± 0.92	0.3554
MBL^4^ at the 2-year follow-up	0.23 ± 0.11	0.18 ± 0.12	0.3339

^1^ SD = Standard deviation. ^2^ NA = Not applicable. ^3^ PES= Pink Esthetic Score. ^4^ MBL=Marginal Bone Loss.

## Data Availability

Additional data are available on request through the corresponding author.
